# On Paralysis from Brain Disease

**Published:** 1875-11

**Authors:** 


*On Paralysis from Brain Disease in its Common Forms.* By H. Charlton Bastian, M. A., M. D., F. R. S.; Illustrated. New York: D. Appleton & Co., 1875. Buffalo: Herger & Ulbrich.

This work consists of a careful revision of lectures which were delivered in the University College Hospital, and published in the *Lancet*. The lectures are eight in number, and treat of the common forms of Paralysis, the result of Brain Disease. The lectures thus collected are an exceedingly clear and readable account of the present condition of knowledge of a subject upon which general practitioners, are from neceesity, but slightly informed. The lectures upon the appoplectic condition, the difficulty of diagnosis between it and certain injuries, coma, from poisoning, intoxication, epilepsy or uræmia; and the remarks upon the differential diagnosis of Embolism, Thrombosis, and Hæmorrhage, are full of interest, but not more so than several other sections of the work. The author hurries over the subject of Electricity in a few words, which fact is to be regretted, for his observations have doubtless led him to form an unfavorable opinion of the value of this agent, and his reasons therefor would have been of interest. The work is full of interest, is well written, and will be an addition to the library of any Physician.

